# Low-Dose Naltrexone for Severe Fibromyalgia Syndrome: A Report of a Case With Two-Year Follow-Up

**DOI:** 10.7759/cureus.83824

**Published:** 2025-05-10

**Authors:** Ulrich Moser

**Affiliations:** 1 Pain Management, German Pain Association, Mönchberg, DEU

**Keywords:** fatigue, fibromyalgia, neurology, neurophysiology, pain management, pharmacology, sleep disorder, widespread pain

## Abstract

Fibromyalgia syndrome (FMS) is characterized by diffuse musculoskeletal pain associated with daytime fatigue, sleep disturbance, cognitive deficits, and often further somatic symptoms. While some patients with FMS respond to standard treatment with amitriptyline, pregabalin, or duloxetine in combination with outpatient multimodal pain management, there are still many who do not benefit sufficiently from this treatment or suffer intolerable side effects. Effective treatment options are therefore needed to supplement conventional therapies. Naltrexone is used in many countries as an off-label therapy in low doses for several chronic immunomodulatory disorders, including FMS. However, the strength of evidence from previous randomized controlled trials is low. I report a patient with severe FMS who did not respond to conventional therapy. Instead, low-dose naltrexone (LDN) (4.5 milligrams per day) resulted in a significant and sustained improvement in most FMS symptoms. The results of this case report suggest that an off-label use of LDN in severe refractory FMS may be a viable option. However, the information base is currently limited, and studies are conflicting.

## Introduction

Fibromyalgia syndrome (FMS) is a chronic, centralized, and generalized pain syndrome with a strong female predilection, characterized primarily by widespread musculoskeletal pain, fatigue, non-restorative sleep, mood disorders, and cognitive impairment. There is a wide range of somatic and psychological symptoms that contribute to significant symptom burden and functional impairment. The prevalence of FMS is estimated to be 1-5% in the general population, depending on the diagnostic criteria used. Despite the high prevalence of FMS, it often takes two years or more for a diagnosis to be made. Established screening criteria are still rarely used in clinical practice, and clinicians often rely on subjective or experiential criteria to decide whether someone has FMS or not [1,2]. FMS alone or in combination with comorbidities such as rheumatic diseases, psychiatric disorders, chronic fatigue syndrome, and sleep disorders poses a challenge to pain practitioners, not only in terms of diagnosis, but also in terms of uncertainty about the efficacy of different pain treatment options [1,2].

A growing body of evidence supports the hypothesis that biological and immune-mediated mechanisms may be central to explaining the symptoms associated with FMS. This has the potential to offer new avenues for therapeutic intervention [2]. Recently, there has been a shift in focus toward the concept of central sensitization as a central pathophysiological process. This suggests that an increase in central and peripheral pain perception and sensory processing within the ascending and descending sensory pathways may be a key factor in disease development [2]. The above findings, combined with the presence of abnormalities on brain imaging in the absence of known peripheral pain generators, have led to the suggestion that the central nervous system may serve as the primary source of pain associated with fibromyalgia [2]. Recent research has demonstrated the possible involvement of an underlying neuroinflammatory process in the pathogenesis of central sensitization in FMS [2]. A number of immune cells, including macrophages, glial cells, monocytes, mast cells, and neutrophils, have been implicated in the development of this inflammatory substrate in FMS [3]. Activation of toll-like receptor 4 in microglia and central nervous system neurons has been shown to facilitate the release of pro-inflammatory cytokines, which have been shown to mediate neuropathic pain. These pro-inflammatory cytokines serve to increase the excitatory tone of the neuronal networks associated with the perception of pain. This can lead to increased pain perception, as well as fatigue, cognitive impairment, and mood and sleep disturbances [4]. In light of these findings, the concept of neuroplastic pain has been proposed as a means of characterizing this chronic pain disorder, which has not previously been encompassed by the pain entities of nociceptive and neuropathic pain [5].

The management of FMS is a major challenge for everyone involved, from the patients and their families to the healthcare professionals responsible for their care. The approach is multidisciplinary and includes physical, psychosocial, and pharmacological interventions [1]. Non-pharmacological therapies, including low-intensity aerobic exercise and psychological techniques, such as pain management training, should be considered as primary interventions. Psychiatric-psychotherapeutic treatment is recommended for psychological comorbidity [1]. Pharmacological treatment (amitriptyline, duloxetine, pregabalin, tramadol) aims to reduce pain and comorbidities such as depression, anxiety, fatigue, and pain [1]. A discrepancy between patient views and guideline recommendations is evident when pharmacological therapies for fibromyalgia are evaluated using patient surveys. A significant number of people with FMS feel that the medications recommended in the guidelines are inadequate or that they are ineffective [2]. In fact, there is evidence that the currently recommended first-line drugs have limited efficacy due to adverse effects, minimal benefit over placebo, and no improvement in patient fatigue or quality of life [6]. Therefore, there is an urgent need for further research into alternative medications that may prove safe and effective for people with FMS.

Naltrexone is an opiate receptor antagonist at a dose of 50 mg, but at lower doses of 1 mg to 4.5 mg, it appears to have analgesic and immunomodulatory effects. Low-dose naltrexone (LDN) was originally introduced into clinical practice in the 1980s at doses of 1.5 mg to 3 mg as an alternative option for a variety of autoimmune diseases [7].

The potential analgesic effect of LDN results from blockade of mu-, delta-, and, to a lesser extent, kappa-opioid receptors in the central nervous system. This appears to lead to a feedback increase in these receptors and an up-regulation of the endorphin system. This "opioid rebound" effect could have several beneficial effects on health and quality of life, including enhanced endogenous analgesia and suppression of critical immune factors [7]. The anti-inflammatory effect of LDN appears to be due to blockade of toll-like receptor 4 on microglial cells in the central nervous system, antagonism of the opioid growth factor receptor, immunomodulation of macrophages and microglia, inhibition of T and B lymphocytes, and other as yet unknown mechanisms. Younger et al. [8] argue that microglial cells may produce a number of pro-inflammatory cytokines, substance P, nitrogen, nitric oxide, and excitatory amino acids when chronically stimulated, as is suspected in FMS. De Carvalho et al. [9] postulate that these substances are associated with what is commonly referred to as sickness behavior: cognitive impairment, mood and sleep disturbances, fatigue, and increased pain and discomfort, symptoms similar to those experienced by people with FMS.

## Case presentation

A woman in her early 40s, measuring 170 centimeters tall and weighing 155 kilograms, presented with multiple shoulder and neck complaints that began approximately six years ago, coinciding with a diagnosis of pulmonary sarcoidosis. Her medical history included class III obesity, arterial hypertension, and obstructive sleep apnea syndrome, for which she utilizes intermittent nocturnal positive pressure ventilation. Additionally, she had insulin-dependent diabetes mellitus, with her glycosylated hemoglobin (HbA1c) levels maintained between 6.5% and 6.8%. At the time of her initial evaluation, her baseline pharmacotherapy included prednisolone at a dosage of 5 mg per day, methotrexate at 15 mg per week, subcutaneous insulin, folic acid, vitamin D, xipamide, lercanidipine, and beclomethasone dipropionate delivered via auto inhaler at a strength of 100 micrograms (µg). Magnetic resonance imaging (MRI) of the cervical spine revealed a herniated disc at the cervical level C6-C7; however, a neurological assessment did not demonstrate any sensory or motor deficits. Over the following months, the patient’s pain exhibited a spreading nature throughout her body, characterized by variable topography. She described the discomfort as a burning sensation in her hands, akin to “a thousand pinpricks” in her feet, likening it to “scratching the skin with a spoon down to the bone.” Despite these significant complaints, the neurological examination, including nerve conduction velocity studies, indicated no evidence of peripheral neuropathy. Furthermore, comprehensive evaluations revealed no spinal or joint pathology during active or passive movements. The patient rated her pain intensity on the Numerical Rating Scale (NRS) [10] as 60-70 at baseline, escalating to 90 at peak episodes (0 = no pain, 100 = worst pain imaginable). On days when her condition was relatively stable, she noted the average pain level at 30-40. Notably, her condition worsened with physical activity, while rest and distraction provided some relief. She reported a lack of pain control, significant disruption of her sleep patterns, and persistent daytime fatigue. In an attempt to manage her symptoms, the patient underwent various therapeutic interventions, including aerobic exercise, manual therapy, psychological counseling, breathing exercises, meditation, and a five-week inpatient rehabilitation program. Additionally, she received three weeks of inpatient multimodal university treatment, which included physical training, physiotherapy, psychotherapy, relaxation techniques, heat application, transcutaneous electrical nerve stimulation, progressive muscle relaxation, and pain management training. Analgesics such as metamizole, ibuprofen, and paracetamol were prescribed alongside amitriptyline at a dosage of 50 mg in the evening. However, these interventions did not yield the desired therapeutic outcomes. The differential diagnosis of FMS is extensive and includes hypothyroidism, hyperparathyroidism, chronic fatigue syndrome, systemic lupus erythematosus, anemia, inflammatory myopathy, polymyalgia rheumatica, osteomalacia, and psychiatric disorders (e.g., post-traumatic stress disorder, anxiety and depression, and sleep disorders). The aforementioned conditions can exhibit symptoms such as pain, fatigue, sleep disorders, and cognitive dysfunction that are also typical of FMS. Nonetheless, the diagnosis of FMS and differentiation from other potential diagnoses can typically be established through a comprehensive history, physical examination, and a limited array of laboratory tests. Possible alternative diagnoses were ruled out in this case by clinical, radiological, and laboratory evaluation.

**Figure 1:**
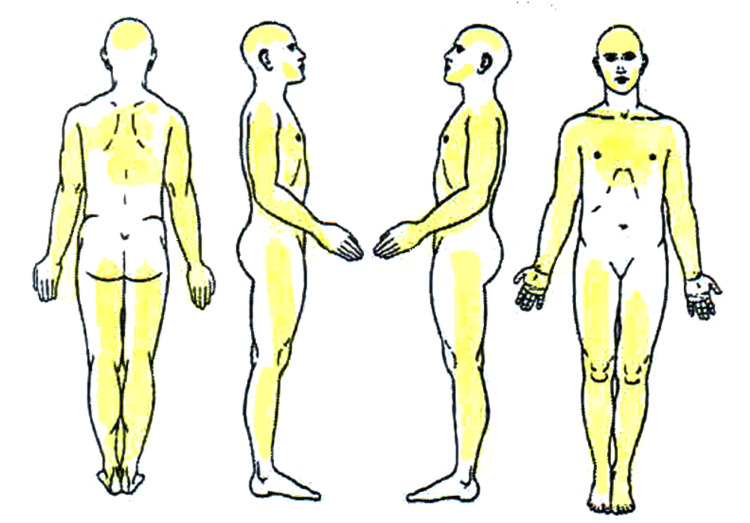
Widespread Pain Index of the patient. At the initial assessment, painful areas were the left shoulder, right shoulder, left arm, right arm, left forearm, right forearm, neck, upper back, left hip, right hip, left thigh, right thigh, left leg, right leg, left jaw, right jaw, and chest. Areas not included were the abdomen and lower back. The Widespread Pain Index (WPI) was 17/19, indicating a high prevalence of pain across multiple body regions. The figure is a free drawing, filled in by the patient to mark the areas of pain.

After several years of unresolved symptoms, the patient’s primary care physician suspected FMS in 2023 and subsequently referred her to a pain specialist. Upon evaluation, the revised American College of Rheumatology criteria [11] were utilized to establish a diagnosis of fibromyalgia with severe somatic impairment. The Widespread Pain Index score was 17 out of a possible 19, indicating a high prevalence of widespread pain (Figure 1).

**Figure 2:**
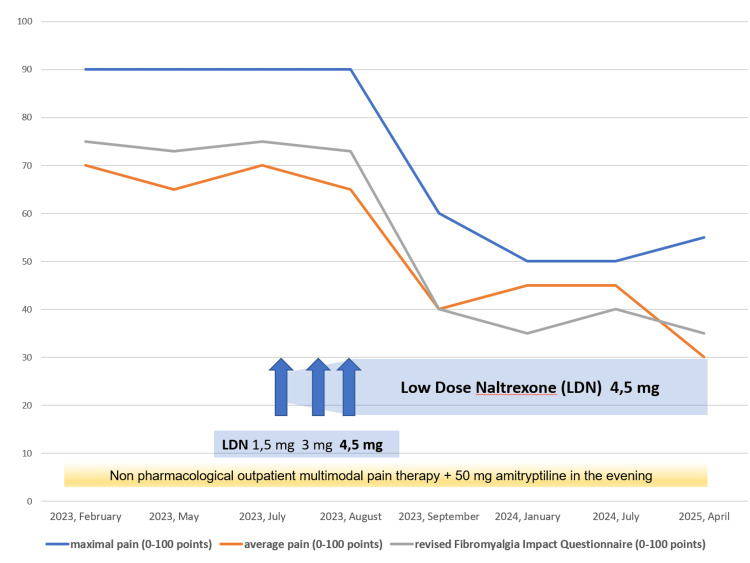
Pain intensity and Fibromyalgia Impact Questionnaire (FIQR) follow-up: February 2023 to April 2025. The revised Fibromyalgia Impact Questionnaire (FIQR, grey line) is a specific questionnaire designed to quantify the disability caused by fibromyalgia syndrome (FMS). A comprehensive analysis of the data from February to August 2023 revealed a value exceeding 70 points (severe impairment). Following the administration of low-dose naltrexone (LDN) 4.5 mg, a decline of 35 points was observed in April 2025, indicating mild severity. The maximum level of pain (blue line) was reduced from a mean of 90 to 55 on the 0-100 Numeric Rating Scale, while the mean level of pain (orange line) decreased from 70 to 30.

The baseline total symptom severity score for FMS was nine out of 12 points, indicating a severe, pervasive, and continuous condition that significantly interferes with daily life. Within the symptomatology, the patient rated fatigue, unrefreshing awakening, and cognitive issues as moderate, assigning two points to each. In the somatic stress assessment, the patient identified 26 additional symptoms, surpassing the threshold of 25 symptoms, which was allocated a score of three points and classified as “severe somatic stress.” Utilizing the revised Fibromyalgia Impact Questionnaire (FIQR) [12], the patient attained a total score of 75 out of a maximum of 100 points (Appendix). The identified FIQR cut-off scores are as follows: remission is defined as ≤30 points; mild severity is >30 and ≤45; moderate severity is >46 and ≤65; and high severity is >65. Therefore, the score of 75 points in this case signifies severe impairment resulting from FMS. The psychological impact of FMS was assessed using the Patient Health Questionnaire for Depression and Anxiety (PHQ-4) [13], which yielded a total score of seven out of 12, indicating moderate psychological impairment (Appendix). Consequently, a multimodal outpatient pain management program was initiated, incorporating exercise, aerobic endurance training, individualized and supervised physiotherapy, aqua gym, aqua jogging, thermal baths, and transcutaneous electrical nerve stimulation. The program emphasized regular anaerobic exercise with the objectives of alleviating the patient’s severe obesity and positively influencing diabetes mellitus, sleep apnea, and specific FMS-related symptoms, including unrefreshing awakening and daytime sleepiness. However, by the summer of 2023, the overall situation remained unsatisfactory. The FMS symptom severity score continued to reflect a high nine out of 12 points, and the pain intensity remained comparable to levels before intervention. Following comprehensive counseling regarding alternative treatment options, the patient declined additional antidepressants or calcium modulators due to concerns related to weight gain, along with a rejection of opioids stemming from apprehension about potential addiction. As a result, an off-label prescription for LDN was issued, commencing with an initial dosage of 1.5 mg in the evening, to be increased to 3 mg and subsequently 4.5 mg at two-week intervals. By September 2023, the patient reported a significant and sustained improvement in pain perception and disability associated with FMS at the dosage of 4.5 mg. In contrast, lower doses of 1.5 mg and 3 mg had proven ineffective. These enhancements positively impacted the patient’s overall quality of life, encompassing general health, physical health, mental health, and the ability to perform routine activities. The FIQR is a specific instrument designed to quantify disability associated with FMS, thereby reflecting the quality of life for patients suffering from this condition. Data analysis demonstrated a reduction in the FIQR score from 75 points in February 2023 to 35 points in April 2025, indicating a transition to a mild severity classification. Furthermore, the maximum pain experienced by the patient decreased from a mean of 90 to 55 on the NRS, while the mean pain level dropped from 70 to 30 on the same scale (Figure 2).

**Figure 3:**
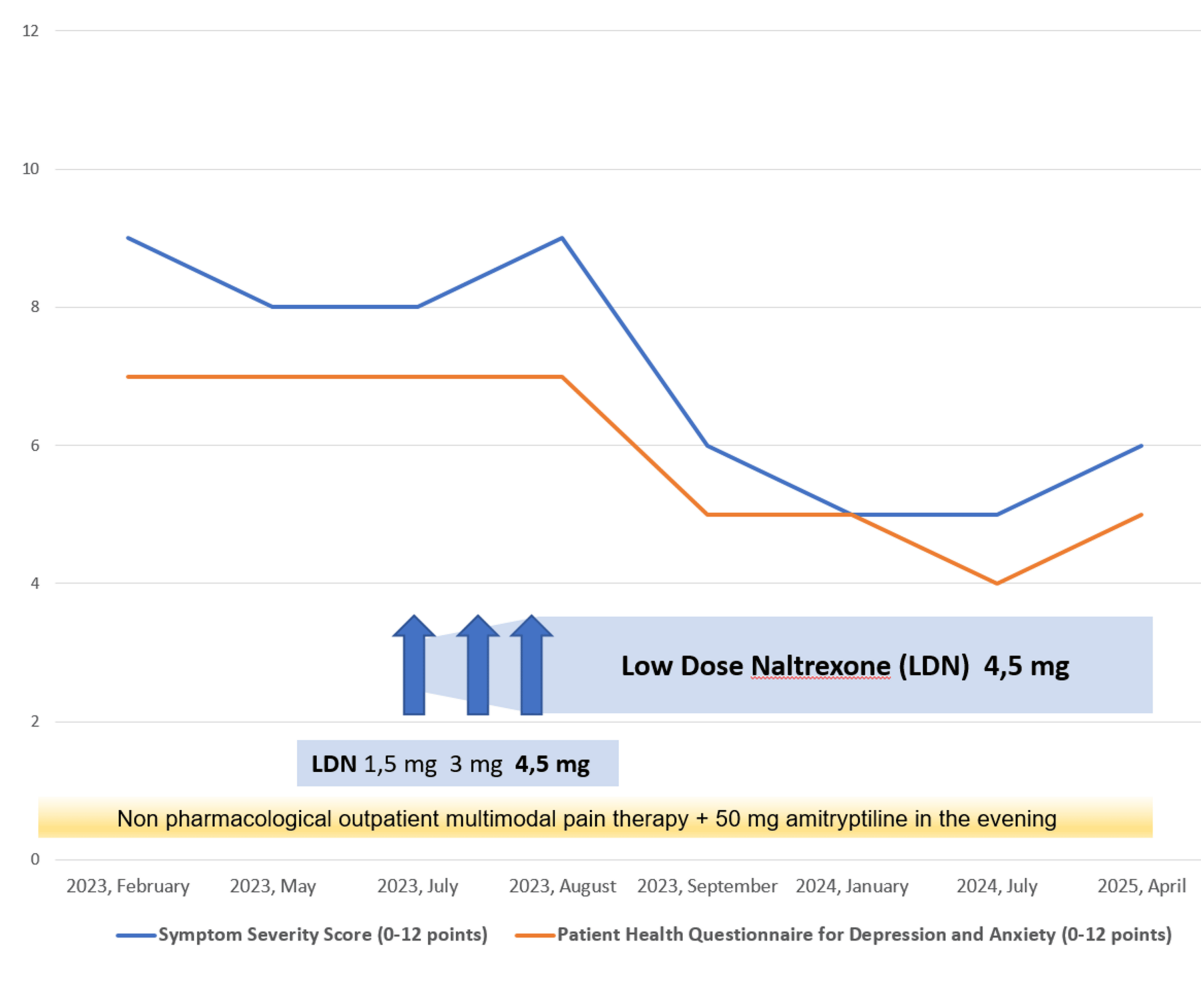
Fibromyalgia severity score and Patient Health Questionnaire for Depression and Anxiety 4 (PHQ-4) follow-up: February 2023 to April 2025. Following the administration of low-dose naltrexone (LDN) 4.5 mg, the total symptom severity score for fibromyalgia syndrome (FMS) (blue line) exhibited a marked improvement, decreasing from nine points on a scale of 12 (severe, pervasive, continuous life-disturbing problems) from February to August 2023 to six points (slight or mild problems) in April 2025. The Patient Health Questionnaire for Depression and Anxiety (PHQ-4, orange line) demonstrated a decline from seven (moderate psychological impairment) to five out of 12 points (mild psychological impairment) over the same time period.

The total symptom severity score for FMS indicated notable improvements in the areas of "waking unrefreshed," "cognitive impairment," and, most significantly, "somatic stress." Notably, the score declined from nine points on a scale of 12 (representing severe, pervasive, and continuous life-disturbing issues) in August 2023 to six points in April 2025, reflecting mild to moderate difficulties. Furthermore, the PHQ-4 demonstrated a significant enhancement in the category of "feeling down, depressed, or hopeless" in September 2023, with stability observed over time. This assessment revealed a reduction from seven points (indicating moderate psychological impairment) to five points out of 12, signifying mild to moderate psychological impairment (Figure 3).

Although the objective of weight loss was not attained, the advantageous effects of LDN on the patients' pain perception and quality of life persist to the present day.

## Discussion

The effectiveness of medications recommended for FMS, such as amitriptyline, milnacipran, duloxetine, gabapentinoids, and tramadol, is limited by a number of factors. These include the occurrence of adverse effects, lack of significant therapeutic benefit compared with placebo, and failure to improve patients' fatigue or quality of life [1]. Given her already morbidly obese status, the patient refused to take any recommended medication other than the existing dose of amitriptyline 50 mg, citing concerns about potential weight gain and addiction. Instead, she agreed to an off-label trial of LDN.

The case presented is a complex one, largely due to the presence of multiple comorbidities that require a comprehensive approach to diagnosis and treatment. In particular, class III obesity has been identified as an independent risk factor for FMS [14]. There is an apparent relationship between physical fitness and FMS, with higher levels of physical fitness being associated with a reduction in the severity of FMS symptoms. In contrast, a reduction in exercise tolerance combined with fatigue may play a role in the low levels of physical activity and increased levels of inactivity and sedentary behavior observed, particularly in obese FMS patients [14]. It is therefore imperative that these patients are offered a therapeutic program that goes beyond simply recommending anaerobic training. In the present case, it is not possible to determine whether the patient was able to implement the recommendation of regular anaerobic training. In addition, no weight loss was achieved.

Regarding other comorbidities, the prevalence of FMS in patients with sarcoidosis, a systemic inflammatory disease of unknown etiology, is about 40% [15]. In patients with diabetes mellitus, the prevalence of fibromyalgia is estimated to be 17-27% [16]. Obstructive sleep apnea syndrome (OSAS) is a disorder of apnea, reduced respiration, and reduced oxygen saturation due to collapse of the upper airway. It is characterized by daytime sleepiness, fatigue, and inattention. It can therefore be postulated that OSAS may also contribute to the patient's symptoms. In addition, approximately 20% of patients with OSAS appear to have FMS [17].

The case illustrates the coexistence of systemic inflammatory comorbidities in the absence of psychiatric illness, with a moderate psychological impact associated with FMS. LDN was shown to relieve pain and improve overall symptoms in this FMS case, including somatic pain, sleep disturbance, and physical and cognitive impairment, although some of these symptoms may have been caused in part by the comorbidities. It is thought that this is primarily due to the anti-inflammatory properties of LDN at a central level, which appear to inhibit the production of several cytokines [7-9,18]. In general, and in this case in particular, FMS does not respond to conventional anti-inflammatory drugs and therefore cannot be considered an inflammatory disease from a traditional perspective [1,2]. However, it is a plausible hypothesis that some degree of inflammation may be present in microglial cells and that cytokine-induced disease behavior, such as that observed in FMS, may result from microglial inflammation [8,9,18].

The patient exhibited no psychiatric comorbidities and demonstrated only moderate psychological symptoms, which may have enhanced the efficacy of LDN. The beneficial effect of LDN in this patient was observed to manifest relatively quickly and has persisted to date. The follow-up period is now more than two years, allowing for the assumption of a stable clinical situation (see Figures 2, 3). The incidence of adverse events has been low in previous studies, with no serious side effects documented [7-9,19]. In this particular case, LDN was also well tolerated.

However, the strength of the evidence from previous randomized controlled trials supporting the use of LDN in people with fibromyalgia appears to be low. This is because previous reviews were conducted without including meta-analyses and were based on case reports, case series, and pilot studies. In addition, most of these reviews did not assess the risk of bias in the included studies. As a result, there is a significant risk of bias that may influence the results of these reviews [7-9,19]. In 2024, a systematic review and meta-analysis was conducted by Vatvani et al. [19], in which only reports with a minimal risk of bias and a double-blind design were selected. Despite the lack of significant results in the individual studies, due to the limited number of subjects and considerable heterogeneity, this meta-analysis clearly demonstrated that the use of LDN significantly and clinically reduces pain levels in people diagnosed with FMS. This finding supports the prescription of LDN in clinical practice as a viable therapeutic option for FMS to help alleviate pain.

The favorable results observed in this case report are consistent with these findings. In this situation, the evidence is not yet clear, and further randomized, placebo-controlled clinical trials may be encouraged by the existence of well-documented case reports.

## Conclusions

The hypothesis that LDN can be used to treat severe refractory FMS in circumstances where conventional use is ineffective, inappropriate, or even contradictory appears to be valid. This hypothesis warrants further investigation. The present case report lends further support to this contention. The findings of this report suggest that LDN has the potential to effectively control pain and improve quality of life in patients with severe refractory FMS, particularly when administered in the setting of systemic inflammation and in the absence of psychiatric comorbidities. However, it is important to note that the existing literature on this subject is still limited. To substantiate this hypothesis, further neurophysiological studies are needed to evaluate the pathophysiology of FMS and the potential of LDN in chronic pain conditions. Furthermore, the identification of factors such as concomitant systemic inflammation and other conditions, including psychiatric comorbidity, may be essential in predicting which patients with FMS will respond to LDN treatment and which will not.

## Appendices

**Table 1:** Maximal and average pain (0-100 points) assessed using revised Fibromyalgia Impact Questionnaire (FIQR) (0-100 points). The maximum level of pain decreased from a mean of 90 to 55 points on the 0-100 Numeric Rating Scale, while the mean level of pain diminished from 65-70 to 30 points from springtime and early summer 2023 to April 2025. The revised Fibromyalgia Impact Questionnaire demonstrates a decline from 75 points in February 2023 to 35 points in April 2025, indicating mild severity at that time.

	Maximal pain (0-100 points)	Average pain (0-100 points)	FIQR
2023, February	90	70	75
2023, May	90	65	73
2023, July	90	70	75
2023, August	90	65	73
2023, September	60	40	40
2024, January	50	45	35
2024, July	50	45	40
2025, April	55	30	35

**Table 2:** Symptom severity score (0-12 points) and Patient Health Questionnaire for Depression and Anxiety (PHQ-4) (0-12 points). The total symptom severity score for fibromyalgia syndrome (FMS) exhibited a decline from nine points on a scale of 12 (severe, pervasive, continuous life-disturbing problems) in August 2023 to six points in April 2025, indicating mild to moderate problems. The Patient Health Questionnaire for Depression and Anxiety showed a decrease from seven (indicating moderate psychological impairment) in August 2023 to five out of 12 points in April 2025, indicating mild to moderate psychological impairment.

		Symptom severity score (0-12 points)	PHQ-4 (0-12 points)
2023, February		9	7
2023, May		8	7
2023, July		8	7
2023, August		9	7
2023, September		6	5
2024, January		5	5
2024, July		5	4
2025, April		6	5
